# Ellagic Acid Resensitizes Gemcitabine-Resistant Bladder Cancer Cells by Inhibiting Epithelial-Mesenchymal Transition and Gemcitabine Transporters

**DOI:** 10.3390/cancers13092032

**Published:** 2021-04-22

**Authors:** Ying-Si Wu, Jar-Yi Ho, Cheng-Ping Yu, Chun-Jung Cho, Chia-Lun Wu, Cheng-Shuo Huang, Hong-Wei Gao, Dah-Shyong Yu

**Affiliations:** 1 Graduate Institute of Pathology and Parasitology, National Defense Medical Center, Taipei 114, Taiwan; wuqiqi850119577@gmail.com (Y.-S.W.); jaryiho@gmail.com (J.-Y.H.); cpyupath@yahoo.com.tw (C.-P.Y.); tomato925929@gmail.com (C.-J.C.); s0915642@gmail.com (C.-L.W.); qo4m4443151@gmail.com (C.-S.H.); 2 Graduate Institute of Life Sciences, National Defense Medical Center, Taipei 114, Taiwan; 3 Department of Pathology, Tri-Service General Hospital, National Defense Medical Center, Taipei 114, Taiwan; 4 Division of Urology, Department of Surgery, Tri-Service General Hospital, National Defense Medical Center, Taipei 114, Taiwan

**Keywords:** ellagic acid, gemcitabine resistance, bladder cancer, TGF-β/Smad signaling pathway, hCNT1 and hENT1

## Abstract

**Simple Summary:**

Chemoresistance of bladder cancer has become a major obstacle to clinical treatment, especially in first-line treatments involving gemcitabine (GCB). Epithelial-mesenchymal transition (EMT) is highly correlated with GCB resistance but less correlated with GCB metabolism and less reported as a novel therapeutic strategy. Our findings indicated that EMT-related GCB resistance occurs through the TGF-β/Smad signaling pathways and involves repressed expression of the GCB transporters hCNT1 and hENT1. Ellagic acid (EA) combined with GCB intensified the chemosensitivity of GCB in resistant cells by repressing Smad2, Smad3, and Smad4 expression and rescuing hCNT1 and hENT transcription. These data suggest that EA is a good adjuvant agent for blocking TGF-β/Smad signaling-related GCB resistance in bladder cancer.

**Abstract:**

Gemcitabine (GCB) resistance is a major issue in bladder cancer chemoresistance, but its underlying mechanism has not been determined. Epithelial-mesenchymal transition (EMT) has been shown to be comprehensively involved in GCB resistance in several other cancer types, but the direct connection between EMT and GCB remains unclear. This study was designed to elucidate the mechanism of EMT-related GCB resistance in bladder cancer and identify a potential phytochemical to modulate drug sensitivity. The biological effects of ellagic acid (EA) or its combined effects with GCB were compared in GCB-resistant cells and the GCB-sensitive line in terms of cell viability, apoptosis, motility, and in vivo tumorigenicity. The molecular regulation of EMT-related GCB resistance was evaluated at both the mRNA and protein expression levels. Our results indicated that TGF-β/Smad induced the overactivation of EMT in GCB-resistant cells and reduced the expression of GCB influx transporters (hCNT1 and hENT1). Moreover, ellagic acid (EA) inhibited the TGF-β signaling pathway both in vitro and in vivo by reducing Smad2, Smad3, and Smad4 expression and thereby resensitized GCB sensitivity. In conclusion, our results demonstrate that TGF-β/Smad-induced EMT contributes to GCB resistance in bladder cancer by reducing GCB influx and also elucidate the novel mechanisms of EA-mediated inhibition of TGF-β/Smad-induced EMT to overcome GCB resistance. Our study warrants further investigation of EA as an effective therapeutic adjuvant agent for overcoming GCB resistance in bladder cancer.

## 1. Introduction

Bladder cancer is the seventh most common cancer worldwide and the second most common urological malignancy. Although patients with superficial tumors initially respond well to transurethral resection and intravesical chemotherapy or immune therapy, approximately 60% of patients ultimately experience local recurrence, and up to 40% of patients progress to invasive metastatic disease with potential lethality [1,2]. The combined treatment of gemcitabine (GCB) and platinum analogs, such as cisplatin and carboplatin, is the standard first-line chemotherapy for patients with locally advanced and metastatic bladder cancer [3,4], but the nephrotoxicity of platinum analogs has become the major dose-limiting side effect [5]. However, recurrence occurs frequently after tumor muscle invasion or distant metastasis, which results in a poor clinical outcome and prognosis, with a median overall survival of less than 14 months [3]. Currently, it is considered urgent to develop alternative remedies to overcome the resistance of bladder cancer to traditional treatment.

Natural compounds or phytochemicals are inexpensive, have high bioavailability and limited toxicity compared with synthetic pharmaceutical agents, which makes them attractive for the field of rationale drug discovery [6]. GCB is a deoxycytidine nucleoside analog with two fluorine atoms inserted into the deoxyribose ring, and metabolites of GCB mask DNA chain termination and extensively inhibit several cell replication enzymes, resulting in highly efficient cytotoxicity for a broad spectrum of solid tumors [7]. The use of phytochemicals participating in anti-DNA replication and overcoming resistance to nucleoside analogs should be a proper alternative treatment strategy to overcome GCB resistance in bladder cancer. Ellagic acid (EA), a polyphenolic compound found as a naturally occurring hydrolysis product of ellagitannins in pomegranates, berries, grapes, green tea, and nuts, was selected as a study candidate for this study not only because of its comprehensive involvement in several antitumor and anti-inflammatory processes [8] but also because of its potential to enhance the sensitivity of nucleoside analogs, as shown in several virological [9,10,11,12] and cancer treatment studies [13].

Despite several signaling dysregulations have been shown to be involved in GCB resistance [14], it is evident that epithelial-mesenchymal transition (EMT) drives the chemoresistance of several cancer cell lines, especially GCB resistance in pancreatic cancer [15,16,17]. However, the role of EMT in GCB resistance in bladder cancer has rarely been addressed, and the molecular events of EMT resulting in the acquisition of chemoresistance are still unclear.

In addition, the dysregulation of several GCB metabolic enzymes or GCB transporter proteins also contributes to GCB resistance, including the inhibition of GCB-activating enzymes such as deoxycytidine kinase (dCK) and thymidine kinase 2 (TK2), upregulation of GCB-inactivating enzymes or substrates such as cytidine deaminase or ribonucleotide reductase M1 or M2, and deficiency of equilibrative nucleoside transporters (hENTs) or concentrating nucleoside transporters (hCNTs) [14,18,19]. However, the direct molecular events underpinning the EMT-induced acquisition of GCB resistance remain unclear. This study was designed to describe the EMT-related regulation of GCB metabolic or transporting proteins in GCB-resistant bladder cancer cells and to evaluate the therapeutic potential of EA combined treatment to overcome GCB resistance.

## 2. Results

### 2.1. EA induces High Cytotoxicity of Gemcitabine in GCB-Resistant Cells

**Figure 1:**
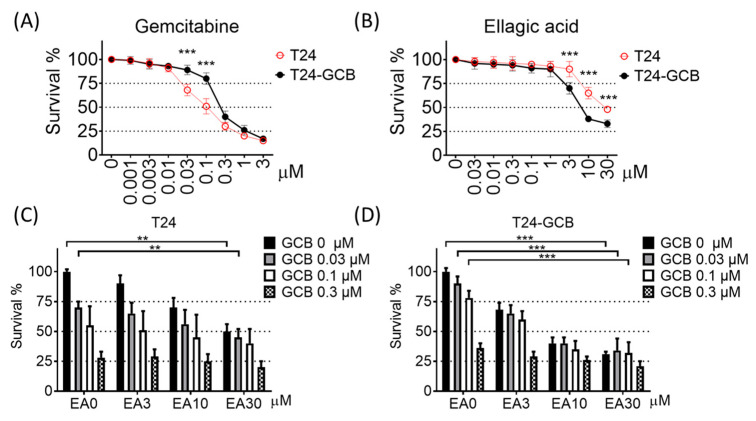
Cytotoxicity was measured with the MTT assay after 48 h of treatment in T24 cells or gemcitabine-resistant T24 cells (T24-GCB) with serial concentrations of (**A**) gemcitabine (GCB) and (**B**) ellagic acid (EA). The cytotoxicity of the combined treatment of EA and GCB was also evaluated in (**C**) T24 and (**D**) T24-GCB cells. ** < 0.01 and *** < 0.001.

To evaluate the GCB resistance of bladder cancer cells, GCB-resistant T24 cells were obtained from long-term maintenance under GCB stress for more than 1 year. As shown in Figure 1B, one strain of GCB-resistant T24 cells (T24-GCB) tolerated gemcitabine; that is, the inhibitory concentration of half survival (IC_50_) was 0.1 µM GCB for T24 parental cells but ~0.3 µM GCB for T24-GCB cells (Figure 1A,B). In addition, the chemosensitivities of T24-GCB cells to mitomycin C, doxorubicin, or cisplatin were similar to those of most bladder cancer cell lines (Supplementary Figure S1B–D). Therefore, we obtained GCB-resistant cells that were more tolerant to GCB than other chemotherapeutic compounds.

Moreover, EA induced significantly higher cytotoxicity in T24-GCB cells than in T24 cells (Figure 1A), and the cytotoxic effect was much higher when EA was combined with GCB in T24-GCB cells (Figure 1C,D). In addition, a similar combined cytotoxic effect was not observed for EA combined with mitomycin C (Supplementary Figure S1E,F). Moreover, the J82 cell, an intrinsically higher GCB-tolerant cell line (Supplementary Figure S1A), was also recruited to evaluate the treatment effects of EA combined with GCB. Similar to the T24-GCB cells, EA also induced higher cytotoxic effects under combination with GCB in the J82 cells than T24 cells (Supplementary Figure S1G,H). Besides, the synergistic effects of the combined treatment of EA and GCB were evaluated in T24, T24-GCB, and J82 cells. The synergism was evaluated with isobologram method using calcusyn software, and the combined treated effects located below the additive line meant there might be a synergistic effect between those two drugs. Further, cisplatin, another standard first-line chemotherapeutic drug of bladder cancer, was selected as another combination and revealed no synergism with EA (Supplementary Figure S2).

### 2.2. EA Combined with GCB Treatment Increases Apoptosis and Reduces Cell Motility in GCB Resistant Cells

**Figure 2:**
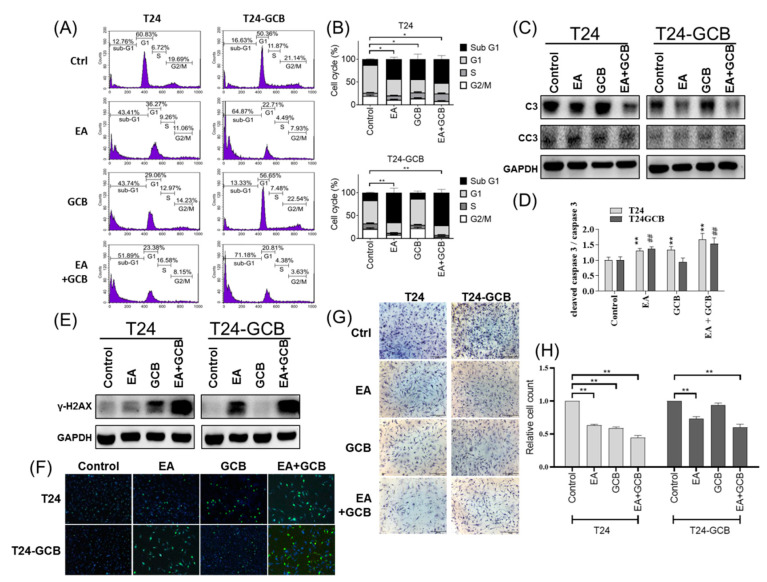
(**A**) Comparison of the cell cycle distribution of T24 and T24-GCB cells among the PBS control, EA (30 µM), GCB (0.1 µM) and EA (30 µM) +GCB (0.1 µM) groups. (**B**) Quantitative bar chart of the cell cycle distribution among the treated groups. (**C**) Protein expression levels of procaspase 3 (C3) and cleaved caspase 3 (CC3) compared among the same groups. (**D**) Quantitative bar chart of C3 and CC3 expression among the treated groups. (**E**) Protein levels of γ-H2AX, a DNA double-strand breakage marker, compared among the same groups. (**F**) Results of the γ-H2AX foci assay compared among the same groups. (**G**) Results of the Matrigel-coated Transwell invasion assays compared among the same groups. (**H**) Quantitative bar chart of migrated cells among the treated groups. All statistical tests were analyzed with Student’s t test with significance at * < 0.05, ** < 0.01 (or ## < 0.001).

As shown in Figure 1A,B, the IC_50_ of 48 h treatment of EA or GCB for T24 cells were 30 µM and 0.1 µM, respectively. All subsequent analyses of biological effects or molecular regulations were also evaluated with 30 µM EA and/or 0.1 µM GCB under 48 h treatment, except the wound-healing assay was compared at 24 h treatment. The combined effect of EA and GCB on cell survival was evaluated under EA treatment with or without GCB by flow cytometry analysis. As shown in Figure 2A,B, either EA or GCB significantly induced a high sub-G1 cell population in T24 cells, which reflect apoptotic cells, and EA combined with GCB induced a much higher sub-G1 population in T24 cells. Additionally, EA with or without GCB induced a higher apoptotic cell population in T24-GCB cells than in T24 cells. Similarly, EA, GCB or EA combined with GCB induced significantly higher cleavage of caspase 3, which functions as the dominant executor of apoptosis, in T24-GCB cells than in T24 cells, especially EA combined with GCB (Figure 2C,D). Moreover, EA also induced higher γ-H2AX levels, which indicate DNA breakage, in T24-GCB cells than in T24 cells, especially in combination with GCB, and thereby restored the DNA damage capacity of GCB in T24-GCB cells (Figure 2E,F).

Moreover, the combined effect of EA and GCB on cell motility was further evaluated with wound healing and Matrigel-coated Transwell invasion assays. EA reduced cell migration (Supplementary Figure S3) and invasion (Figure 2G,H), and EA combined with GCB resulted in a more efficient inhibition of cell motility in both T24 and T24-GCB cells than EA alone. Moreover, similar to the T24-GCB cells, higher cell viability (Supplementary Figure S4A) and motility (Supplementary Figure S4B,C) were also observed in the J82 cells than T24 cells. EA combined with GCB also induced higher apoptosis in J82 cells including more sub-G1 cell population and more cleaved caspase 3 and also resulted in more DNA damages as higher γ-H2AX detected (Supplementary Figure S4D,E).

### 2.3. EA resensitizes Bladder Cancer Cells to GCB by Reducing Epithelial-Mesenchymal Transition

**Figure 3:**
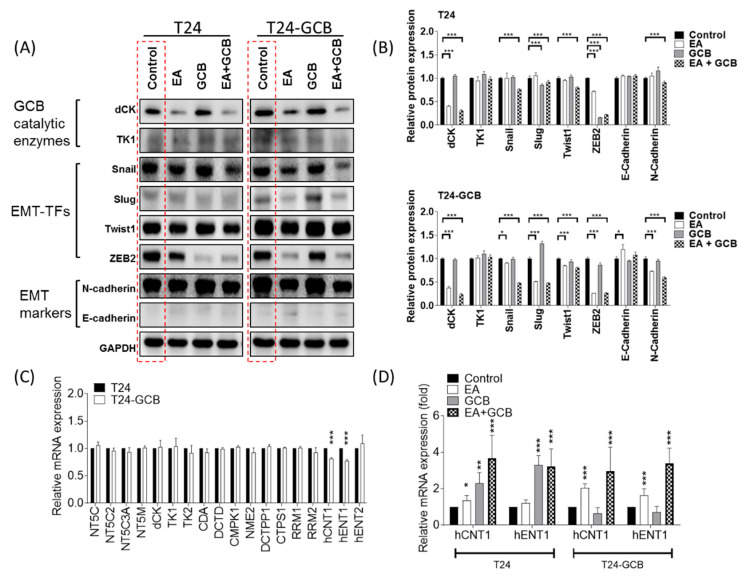
(**A**) Protein expression levels of GCB catalytic enzymes (dCK and TK1), EMT-TFs (Snail, Slug, Twist1 and ZEB2) and EMT markers (N-cadherin and E-cadherin) in T24 and T24-GCB cells were compared among the PBS control, EA (30 µM), GCB (0.1 µM) and EA (30 µM) + GCB (0.1 µM) groups. (**B**) Quantitative bar chart of the protein expression of the indicated EMT-TFs among the treated groups. (**C**) Relative mRNA expression of 15 GCB metabolic enzyme genes and 3 transporter genes compared between T24 and T24-GCB cells. (**D**) Relative mRNA expression of hCNT1 and hENT1 compared among the PBS control, EA, GCB and EA+GCB groups between T24 and T24-GCB cells. All statistical tests were analyzed with Student’s t test with significance at * < 0.05, ** < 0.01 and *** < 0.001.

To analyze the regulatory mechanism of EA in GCB resistance, the major GCB catalytic enzymes deoxycytidine kinase (dCK) and cytosolic thymidine kinase (TK1) were compared. However, no significantly different patterns were found between EA- and/or GCB-treated T24 and T24-GCB cells; that is, EA and EA combined with GCB reduced dCK and TK1 protein expression in both cell lines. This result implied that dCK and TK1 may not be directly attributed to GCB resistance in T24-GCB cells. Since EMT has been shown to be comprehensively involved in GCB resistance in several cancer types, subsequent comparisons were carried out among major transcription factors of EMT (EMT-TFs), including Snail, Slug, Twist1 and ZEB2. Among the EMT-TFs, Slug and Twist1 were remarkably upregulated in T24-GCB cells compared with T24 cells, but Snail and ZEB2 expression was similar between the two cell lines (Figure 3A, the red dashed-line box, quantified in Figure 3B). Interestingly, EA reduced each EMT-TF in both T24 and T24-GCB cells, but a more distinct inhibitory efficacy was observed in T24-GCB cells. Moreover, Slug and ZEB2 were significantly inhibited by GCB in T24 cells but were dramatically refractory in T24-GCB cells, which implied that these two EMT-TFs played some important regulatory roles in GCB resistance. To elucidate the direct connection between EMT and GCB, the mRNA expression levels of 15 GCB metabolic enzymes and 3 major transporters were also evaluated. As shown in Figure 3C, the mRNA expression of hCNT1 and hENT1 was significantly deregulated in T24-GCB cells, and EMT has recently been reported to inhibit hCNT and hENT function in GCB-resistant cancer cells [20,21]. EA or EA combined with GCB upregulated hCNT1 and hENT1 in T24-GCB cells (Figure 3D). These results indicated that EA inhibited the Slug and ZEB2 pathways and might thereby rescue hCNT1 and hENT1 expression and function in GCB-resistant bladder cancer cells. Moreover, EA also inhibited the Slug and ZEB 2 expression (Supplementary Figure S5A,B) in J82 cells and might also thereby rescue hCNT1 and hENT1 expression (Supplementary Figure S5C).

### 2.4. EA Reduces EMT by Inhibiting the TGFβ-SMAD2/3 Upward Signaling Pathway

**Figure 4:**
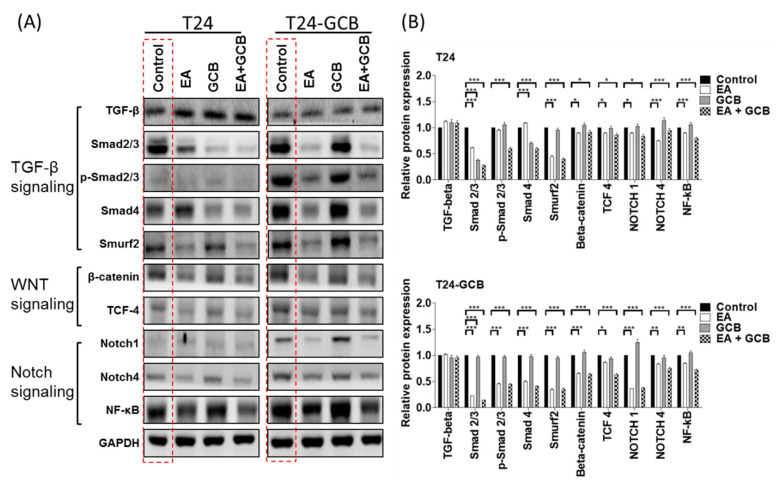
(**A**) Protein expression levels of members of the TGF-β signaling pathway (TGF-β, Smad2/3, p-Smad2/3 and Smad4), WNT signaling pathway (β-catenin and TCF-4) and Notch signaling pathway (Notch1, Notch4 and NF-κB) were compared among the PBS control, EA (30 µM), GCB (0.1 µM) and EA (30 µM) + GCB (0.1 µM) groups between T24 and T24-GCB cells. (**B**) Quantitative bar chart of the protein expression of the indicated members among the treated groups. All statistical tests were analyzed with Student’s test with significance at * < 0.05, ** < 0.01 and *** < 0.001.

To determine the upstream regulation of Slug and ZEB2 in T24-GCB cells, representative members of the three most commonly described EMT inductive signaling pathways, the WNT, Notch, and TGF-β signaling pathways, were compared under treatment with EA with or without GCB. Although most of the representative members of the TGFβ and Notch signaling pathways were significantly upregulated in T24-GCB cells compared with T24 cells (Figure 4A, the red dashed-line box, quantified in Figure 4B), the TGFβ signaling members Smad2, Smad3, and Smad4 were not inhibited by EA in T24 cells but were EA-sensitive in T24-GCB cells, which not only implied that the TGFβ-Smad signaling pathway participates in GCB resistance in T24 cells but also indicated that EA targets this pathway to resensitize GCB in T24-GCB cells. Although several members of the WNT and Notch signaling pathways were also significantly downregulated after treatment with EA or EA combined with GCB, their expression fluctuations were similar between T24 and T24-GCB cells, which indicated that they led to a minor response and played a minor role in GCB resistance (Figure 4). Further, EA also reduced Smad2, Smad3, and Smad4 expression in J82 cells and might thereby inhibited TGF-β/Smad-induced EMT to increase the GCB sensitivity of J82 cells (Supplementary Figure S6).

### 2.5. EA Inhibits the Growth of Bladder Cancer Tumors and Increases the In Vivo Inhibitory Effects of GCB on Tumors

**Figure 5:**
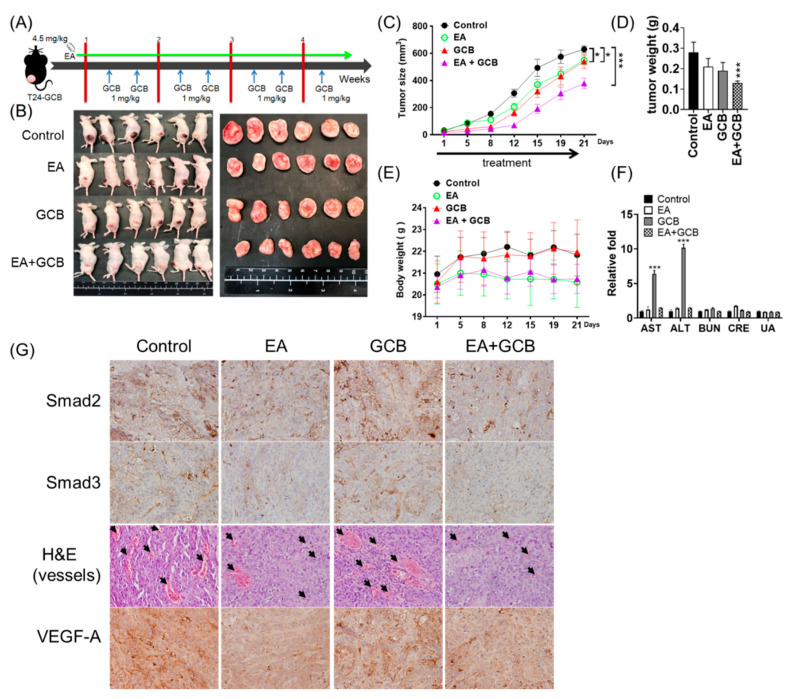
(**A**) Administration procedure of the xenograft and treatment. (**B**) Xenografts of T24-GCB cells were treated with the PBS control, EA (4.5 mg/kg), GCB (1 mg/kg) and EA (4.5 mg/kg) + GCB (1 mg/kg), and in vivo tumors and dissected tumor masses were aligned. (**C**) Curves of tumor size, (**D**) tumor weight, (**E**) mouse body weight and (**F**) liver (AST and ALT) and kidney (BUN, CRE and UA) function among the treated groups. (**G**) Immunohistochemical staining of Smad2, Smad3 and VEGF-A and H&E staining (the black arrows indicate intratumoral vessels) of xenograft tumors among the treated groups. All statistical tests were analyzed with Student’s t test with significance at * < 0.05 and *** < 0.001.

To evaluate the combined effects of EA and GCB on in vivo tumorigenicity, a T24-GCB xenograft mouse model was employed for the comparison of EA, GCB and EA combined with GCB treatments. The administration procedure is illustrated in Figure 5A. After T24-GCB cells were subcutaneously implanted and the tumors were allowed to grow to a diameter of 2 mm, 4.5 mg/kg EA was intraperitoneally injected daily, and 1 mg/kg GCB was intraperitoneally injected twice a week for 4 weeks. Despite either EA or GCB treatment significantly reduced the tumor growth rate compared to the control groups, EA combined with GCB reduced the tumor size by half compared to the control (Figure 5B–D). There was no perceptible influence of body weight or organ toxicity in the treated mice among the different treatment groups (Figure 5E, Supplementary Figure S7C). Moreover, EA also conferred a significant liver-protective effect to reduce GCB-induced hepatotoxicity, albeit without a change in kidney function (Figure 5F).

On a molecular basis, EA or EA combined with GCB obviously reduced the intratumoral expression of Smad 2 and Smad 3, reduced angiogenesis within tumor burden and had inhibitory effects on vascular endothelial growth factor-A (VEGFA), a TGFβ signaling transactivated proangiogenic factor (Figure 5G, Supplementary Figure S7A). Similar inhibitory effects were observed in T24 and T24-GCB cells (Supplementary Figure S7B). These findings provide an explanation for the antiangiogenic ability of EA described in previous studies of several cancer types [22].

## 3. Discussion

GCB resistance is a major type of chemotherapeutic resistance in bladder cancer, and the EMT process has been shown to be comprehensively involved in GCB resistance in several cancer types [21,23,24,25], but it has been less addressed in bladder cancer. There are two novel findings from the present study. First, the TGFβ-Smad2/3-ZEB2-hENT1 axis is specifically involved in EMT-related GCB resistance in bladder cancer cells, including acquired GCB-resistant T24-GCB cells and de novo resistant J82 cells, although members of the β-catenin and Notch signaling pathways are also upregulated in GCB-resistant T24 cells. Second, EA significantly inhibits Smad2 and Smad3 expression both in vitro and in vivo, results in a reduction of Slug and ZEB2 expression and leads to the downregulation of hCNT1 and hENT1.

Activation of the EMT process is comprehensively involved in chemoresistance through a variety of signaling pathways that induce EMT, and several of these signaling pathways have been shown to control both the intrinsic and acquired chemoresistance of GCB in different cancer types, with a majority of them described in pancreatic cancer, including overactivation of the WNT/β-catenin signaling, Notch signaling, hedgehog signaling and TGF-β signaling pathways [14,18,20]. In addition, the induction of GCB resistance by a complicated tumor microenvironment [26] and highly metastatic characteristic is the main cause of chemotherapeutic failure in bladder cancer. Mesenchymal proteins are usually overexpressed and mediate chemoresistance in a variety of cancers [27,28]. Nevertheless, TGF-β signaling-activated EMT is more commonly reported in bladder cancer than other cancers; that is, activation of TGF-β signaling by NUSAP1 has been shown to be negatively correlated with the GCB response in bladder cancer cells [29], and the TGF-β1/Smad signaling-induced lncRNA-LET/miR-145 axis has also been reported to induce gemcitabine resistance [30].

On the other hand, TGF-β signaling-induced EMT-related GCB resistance has also been documented in pancreatic [31], oral [32] and biliary tract [33] cancers. Our findings indicated that TGF-β signaling induced EMT in GCB-resistant bladder cancer cells by activating Smad2/3, thereby upregulating Slug and ZEB2 and ultimately reducing hENT1 and hCNT1, the major influx transporters of GCB [14,18]. Similarly, TGF-β signaling downregulating hENT1 and hCNT3 has also been reported to enhance GCB resistance in pancreatic cancer cells [34]. In addition, EA has been reported to reverse the EMT process both in vitro and in vivo in pancreatic cancer [35,36] and glioblastoma [37]. EA has also been shown to repress the TGF-β signaling pathway in breast cancer [38,39] and prostate cancer cells [40]. Our results suggest that EA contributes to EMT reversion by inhibiting the TGF-β/Smad signaling pathways.

Our results also indicate that EA attenuates the hepatotoxicity induced by GCB [41] in a xenograft model, and similar results have also been found in animals administered several chemotherapeutic agents, including methotrexate [42,43], cyclophosphamide [44] and cisplatin [45,46]. EA liver-protective effects have also been observed for several noncancer drugs or environmental hepatotoxic compounds. GCB is used as the first-line agent for treating several cancer types and has achieved favorable therapeutic effects in bladder cancer and other cancers. However, the severe systemic toxicity caused by a high dose of GCB limits its application and reduces its chemotherapeutic effect. Our findings also implicate that EA combined with GCB may reduce the dormant complications of GCB and that EA may function as an adjuvant therapeutic agent in GCB-related chemotherapy of bladder cancer.

## 4. Materials and Methods

### 4.1. Cell Lines

Four human bladder cancer cell lines, TSGH-8301, TSGH-9202, T24 and J82, were acquired from the American Type Cell Collection or the Bioresource Collection and Research Center. All cells were incubated in RPMI 1640 medium containing 10% fetal bovine serum and 1 µg/mL penicillin and streptomycin (Life Sciences, Palo Alto, CA, USA) at 37 °C in a 5% CO_2_ humidified incubator. Gemcitabine-resistant T24 cells (T24-GCB) were established and maintained in regulatory culture media containing 0.03 μM gemcitabine hydrochloride (Sigma-Aldrich, G6423) for more than 1 year.

### 4.2. Cell Viability Assay

The viability of bladder cancer cells was determined using the 3-[4,5-dimethylthiazol-2-yl]-2,5-diphenyl-tetrazolium bromide (MTT, Sigma-Aldrich, St. Louis, MO, 63103, USA) assay. 3000 cells/well were seeded in 96-well plates and cultured overnight. After treatment under the indicated conditions for 48 h, cells were incubated with 0.1 mg/mL MTT for 3 h, and formazan was dissolved in dimethyl sulfoxide (Sigma-Aldrich, St. Louis, MO, 63103, USA) at room temperature. The absorbance at 560 nm was measured with a spectrophotometer (Bio-Rad Inc., Hercules, CA, USA).

### 4.3. Flow Cytometry

Cells were harvested at the indicated times and fixed in 4% paraformaldehyde (BD Biosciences). After washing twice with ice-cold 1 × PBS, 1 × 10^5^ cells (100 μL) were transferred to a 5 mL culture tube, followed by treatment with 5 μL of propidium iodide with gentle vortexing and a 15-min incubation at room temperature (25 °C) in the dark. Then, 400 μL of 1 × PBS was added to each tube and analyzed by a BD FACSCalibur within 20 min.

### 4.4. Cell Migration and Invasion Assays

In the wound healing assays, 1 × 10^5^ cells were seeded in 6-well plates and incubated to confluence. For each treatment, cells were scraped with a sterile 200 µL pipette tip to generate a clear line in the wells at time 0. The migrated cells were observed under a phase-contrast microscope every 8 h (Leica DMI4000B, Bucks, UK).

Transwell invasion assays were performed using Matrigel-coated 8 µm Transwell chambers (Corning, Steuben County, NY, USA) with 1 × 10^4^ cells in each treatment under incubated in a humidified 5% CO_2_ incubator at 37 °C for 24 h. Cells were then fixed with 4% paraformaldehyde, and the inner surface of the Transwell chambers was wiped with cotton swabs to remove unmigrated cells. After washing, the chambers were stained with crystal violet (Sigma-Aldrich) for 15 min, and the Transwell membranes were torn and kept on slides. Five random fields of each treatment were photographed at 100× magnification. All wound width and invaded cell were measured with ImageJ software.

### 4.5. Western Blotting

Total protein was extracted from cultured cells using RIPA buffer (Thermo Fisher Scientific, Waltham, MA, USA) supplemented with a protease inhibitor cocktail (Roche, Basel, Switzerland) at a ratio of 100:1. The protein concentration was determined by a BCA protein assay kit (Thermo Fisher Scientific, Waltham, USA). Proteins (30 µg) were subjected to 10% SDS-PAGE and then transferred onto a polyvinylidene fluoride (PVDF) membrane (Millipore, Burlington, MA, USA). The membrane was blocked with 5% BSA (Sigma-Aldrich) in TBST (10 mM Tris pH 7.4, 150 mM NaCl, 0.1% Tween-20) and incubated with primary antibodies, as listed in Supplementary Table S1, overnight at 4 °C. Subsequently, the blots were washed with TBST, followed by incubation with an HRP-conjugated goat anti-mouse or goat anti-rabbit secondary antibody (1:5000; Santa Cruz Biotechnology, Dallas, TX, USA) at room temperature for 1 h. The immunoreactive bands were visualized with Immobilon Western Chemiluminescent HRP Substrate (Millipore, Burlington, MA, USA) and analyzed with the UVP GelStudio PLUS System (Analytik Jena AG, Thuringia, Germany). All original western blots were showed in Figure S8.

### 4.6. Quantitative Real-Time PCR

Total RNA was extracted from cells using the TOOLSmart RNA Extractor (BIOTOOLS, Taiwan), and their concentration was measured using a NanoDrop spectrophotometer (Thermo Fisher Scientific). Total RNA was reverse transcribed into cDNA using the ToolsQuant II Fast RT Kit with oligo (dT) primers (BIOTOOLS) in a 20 µL reaction system consisting of 1 µg RNA, 2 µL 10 × RT Reaction Premix with oligo (dT) primers, 1.5 µL ToolsQuant II Fast RT and RNase-free ddH_2_O and incubated at 42 °C for 15 min. Quantitative real-time PCR was performed in a 20 µL reaction system containing 0.1 µg cDNA, 10 µL TOOLS 2X SYBR qPCR Mix (BIOTOOLS) and 5 pmol gene-specific forward and reverse primers in a QuantStudio 5 Real-Time PCR System (Applied Biosystems, Foster City, CA, USA). Supplementary Table S2 lists the primer sequences. After the initial denaturation step at 95 °C for 15 min, amplifications were carried out with 45 cycles at a melting temperature of 95 °C for 15 s, an annealing temperature of 60 °C for 20 s and an elongation temperature of 72 °C for 20 s. The specificity of the amplicons was confirmed by melting curve analysis. GAPDH was used as a reference gene. The relative expression levels of the target genes were calculated using the 2^−ΔΔCT^ method, and no-template controls were included in each run.

### 4.7. Xenograft and Treatment of EA and/or GCB

Female Balb/c nude mice (8 weeks old) were purchased from the National Laboratory Animal Center in Taiwan and acclimated for 1 week. Then, 1 × 10^7^ T24-GCB cells (passage 8) in 100 μL of 1 × PBS with 10 mg/mL Matrigel (BD Biosciences) were implanted into the left flank of each mouse. Before xenografting, cells were tested for mycoplasma using the e-Myco Mycoplasma PCR Detection Kit (Intron). Until the tumors reached a ~20 mm^3^ palpable mass, mice were divided into four groups randomly and intraperitoneally injected with PBS (100 µL), EA (4.5 mg/kg), GCB (1 mg/kg) or EA (4.5 mg/kg) combined with GCB (1 mg/kg). PBS or EA was intraperitoneally injected daily, and GCB was intraperitoneally injected twice a week for 4 weeks. The tumor size was measured every 2 days using the calipers and was calculated by (length × width^2^)/2. Tumors were removed at day 21 to measure tumor weight and were fixed with formalin for immunohistochemistry. Liver and kidney function assays were analyzed with the TMC Service Package (Taiwan Mouse Clinic, Taiwan).

### 4.8. Immunohistochemistry and γ-H2AX Foci Assay

Immunohistochemistry was carried out in formalin-fixed paraffin-embedded xenografted specimens. In brief, 4-μm sections of the xenografted specimen sections were blocked with 10% goat serum for 1 h and incubated with antibodies (1:200) for 2 h at room temperature, washed 3 times with TBST for 10 min followed by Super Sensitive Polymer HRP Detection System/DAB (BioGenex) and counterstained with hematoxylin. Immunohistochemical data were quantified as positivity for each specimen with an Aperio ImageScope and Spectrum Ver. 12.0.

For γ-H2AX foci assay, the prepared cell suspensions were adjusted to the cell density of 5 × 10^5^ cells/mL and subsequently seeded on glass slides overnight. The adherent cells were fixed with 4% paraformaldehyde for 15 min, permeabilized with 0.5% (*v*/*v*) Triton X-100 in PBS for 15 min, blocked with 10% (*v*/*v*) fetal calf serum in PBS at 37 °C for 1-h, incubated for overnight at 4 °C with rabbit monoclonal γ-H2AX (Ser-139) (at a dilution of 1:500, Cell Signaling Technology, Danvers, MA, USA) and incubated with goat anti-rabbit secondary antibody labeled with Alexa Fluor 488 (Molecular probe, Life Technologies, Grand Island, NY, USA) at a 1:500 dilution at room temperature in the dark for 1-h. After counterstained with DAPI (Sigma-Aldrich) and covered with a coverslip, the numbers of green γ-H2AX foci were observed under the Olympus BX51 fluorescence microscope (Tokyo, Japan).

### 4.9. Statistical Analysis

All statistical analyses were performed with SPSS 22.0 (IBM, SPSS, Chicago, IL, USA) and GraphPad Prism 8.0 (GraphPad Software, La Jolla, CA, USA). The data were recorded as continuous variables and analyzed with Student’s t test. The results are expressed as the mean ± standard deviation of at least three separate experiments. All statistical tests and p values were two-sided, and the level of significance was set at < 0.05 (*), < 0.01 (**) or < 0.001 (***).

## 5. Conclusions

**Figure 6:**
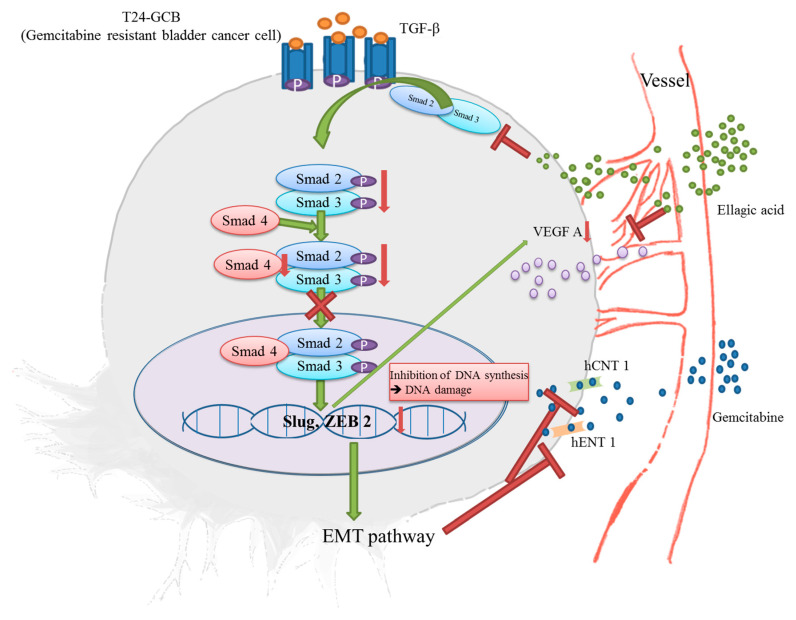
Schematic diagram of EA combined with GCB regulating the EMT process in GCB-resistant bladder cancer cells.

Our results indicate that the TGFβ-Smad2/3 signaling pathway-induced EMT process is involved in GCB resistance in bladder cancer by transactivating Slug and ZEB2 and downregulating hCNT1 and hENT1. Moreover, EA combined with GCB significantly reduces GCB resistance both in vitro and in vivo by inhibiting Smad2/3 expression, attenuating the EMT process and recovering GCB transporters (the schematic diagram of this process is illustrated in Figure 6).

## Data Availability

Data sharing not applicable. No new data were created or analyzed in this study. Data sharing is not applicable to this article.

## Supplementary Materials

The following are available online at https://www.mdpi.com/article/10.3390/cancers13092032/s1, Figure S1: Cytotoxicities of (A) gemcitabine, (B) mitomycin C, (C) doxorubicin and (D) cisplatin compared among different bladder cancer cell lines, including TSGH-8301, TSGH-9202, J82, T24 and T24-GCB. The cytotoxicity of the combined treatment of EA and mitomycin C (MMC) was evaluated in (E) T24 and (F) T24-GCB cells. And the cytotoxicity of the combined treatment of EA and GCB was also evaluated in (G) T24 and (H) J82 cells. All statistical tests were analyzed with Student’s t test with significance at * <0.05, ** <0.01 and *** <0.001, Figure S2: Isobologram analyses of combined effects between ellagic acid (EA) and gemcitabine (GCB) or EA and cisplatin (CIS) were compared in T24, J82 and T24-GCB cells, Figure S3: Cell motility of T24 and T24-GCB cells as determined by the woundhealing assay compared among the PBS control, EA, GCB and EA+GCB groups. Quantitative bar charts of migrated cells in the treated groups were showed on the right side. All statistical tests were analyzed with Student’s t test with significance at ** <0.01 and *** <0.001, Figure S4: (A) Comparison of the cell cycle distribution of T24 and J82 cells among the PBS control, EA (30 µM), GCB (0.1 µM) and EA (30 µM) + GCB (0.1 µM) groups. (B) Results of the Matrigel-coated Transwell invasion assays compared among the same groups. (C) Quantitative bar chart of migrated cells among the treated groups. (D) Protein expression levels of procaspase 3 (C3) and cleaved caspase 3 (CC3) and γH2AX in T24 and J82 cells were compared among the same groups. (E) Quantitative bar chart of C3 and CC3 expression among the treated groups. All statistical tests were analyzed with Student’s t test with significance at ** <0.01 and *** <0.001 (or %%% <0.001), Figure S5: (A) Protein expression levels of EMT-TFs (Snail, Slug, Twist1 and ZEB2) and EMT markers (N-cadherin and E-cadherin) in T24 and J82 cells were compared among the PBS control, EA (30 µM), GCB (0.1 µM) and EA (30 µM) + GCB (0.1 µM) groups. (B) Quantitative bar chart of the protein expression of the indicated EMT-TFs among the treated groups. (C) Relative mRNA expression of hCNT1 and hENT1 compared among the same groups between T24 and J82 cells. All statistical tests were analyzed with Student’s t test with significance at * <0.05, ** <0.01 and *** <0.001, Figure S6: (A) Protein expression levels of members of the TGF-β signaling pathway (TGF-β, Smad2/3, p-Smad2/3, Smad4 and Smurf2) were compared among the PBS control, EA (30 µM), GCB (0.1 µM) and EA (30 µM) + GCB (0.1 µM) groups between T24 and J82 cells. (B) Quantitative bar chart of the protein expression of the indicated members among the treated groups. All statistical tests were analyzed with Student’s t test with significance at * <0.05, ** <0.01 and *** <0.001, Figure S7: (A) Immunohistochemical staining of CD31 and CD34 in xenograft tumors from T24-GCB xenograft mice treated with the PBS control, EA, GCB and EA+GCB. (B) Protein expression levels of VEGFA and VEGFR-2 in T24 and T24-GCB cells compared among the PBS control, EA, GCB and EA+GCB groups. Quantitative bar charts of the expression of the indicated proteins among the treated groups were showed. All statistical tests were analyzed with Student’s *t* test with significance at * <0.05, ** <0.01 and ***<0.001. (C) Alignment of H&E staining of the brain, heart, liver, spleen, lung, kidney, stomach and bladder of xenografted mice that received the four treatments, and no perceptible necrosis was observed, Figure S8: Original Western Blots, Table S1: Lists of the primary antibodies, Table S2: Lists of the primer sequences.
