# Supplementary material for: Ellagic Acid Resensitizes Gemcitabine-Resistant Bladder Cancer Cells by Inhibiting Epithelial-Mesenchymal Transition and Gemcitabine Transporters
Source: Cancers (Basel). 2021 Apr 22;13(9):2032. doi: 10.3390/cancers13092032 (PMC8122772; doi:10.3390/cancers13092032)
Supplement: Supplementary file 1 [file cancers-13-02032-s001.zip › cancers-1197128-suppl-proof_20210425.pdf]

Supplementary Materials: Ellagic Acid Resensitizes Gemcitabine-Resistant Bladder Cancer Cells by Inhibiting Epithelial-Mesenchymal Transition and Gemcitabine Transporters

Ying-Si Wu, Jar-Yi Ho, Cheng-Ping Yu, Chun-Jung Cho, Chia-Lun Wu, Cheng-Shuo Huang, Hong-Wei Gao and Dah-Shyong Yu

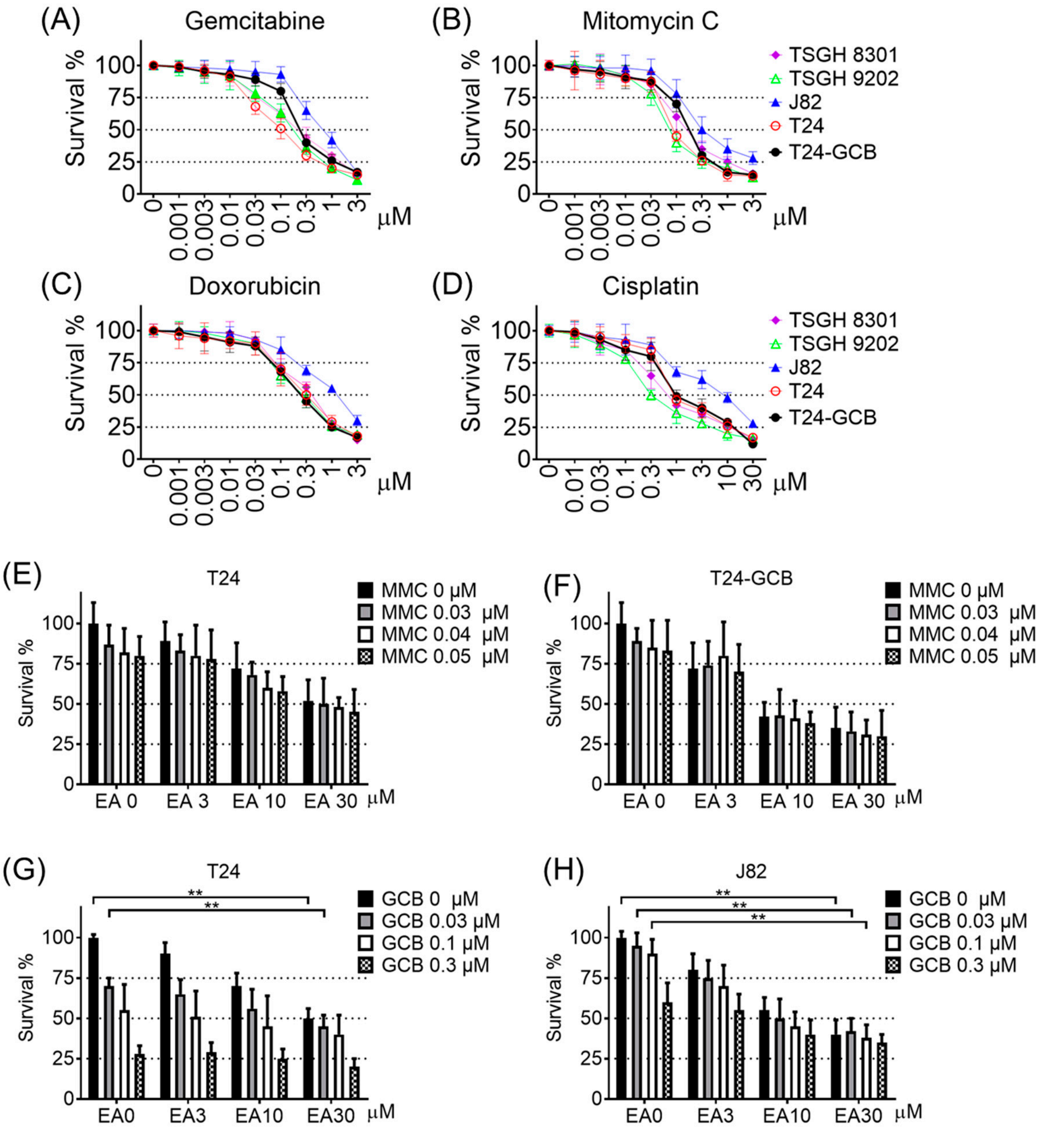

Figure S1. Cytotoxicities of (**A**) gemcitabine, (**B**) mitomycin C, (**C**) doxorubicin and (**D**) cisplatin compared among different bladder cancer cell lines, including TSGH-8301, TSGH-9202, J82, T24 and T24-GCB. The cytotoxicity of the combined treatment of EA and mitomycin C (MMC) was evaluated in (**E**) T24 and (**F**) T24-GCB cells. And the cytotoxicity of the combined treatment of EA and GCB was also evaluated in (**G**) T24 and (**H**) J82 cells. All statistical tests were analyzed with Student's t test with significance at * <0.05, ** <0.01 and *** <0.001.

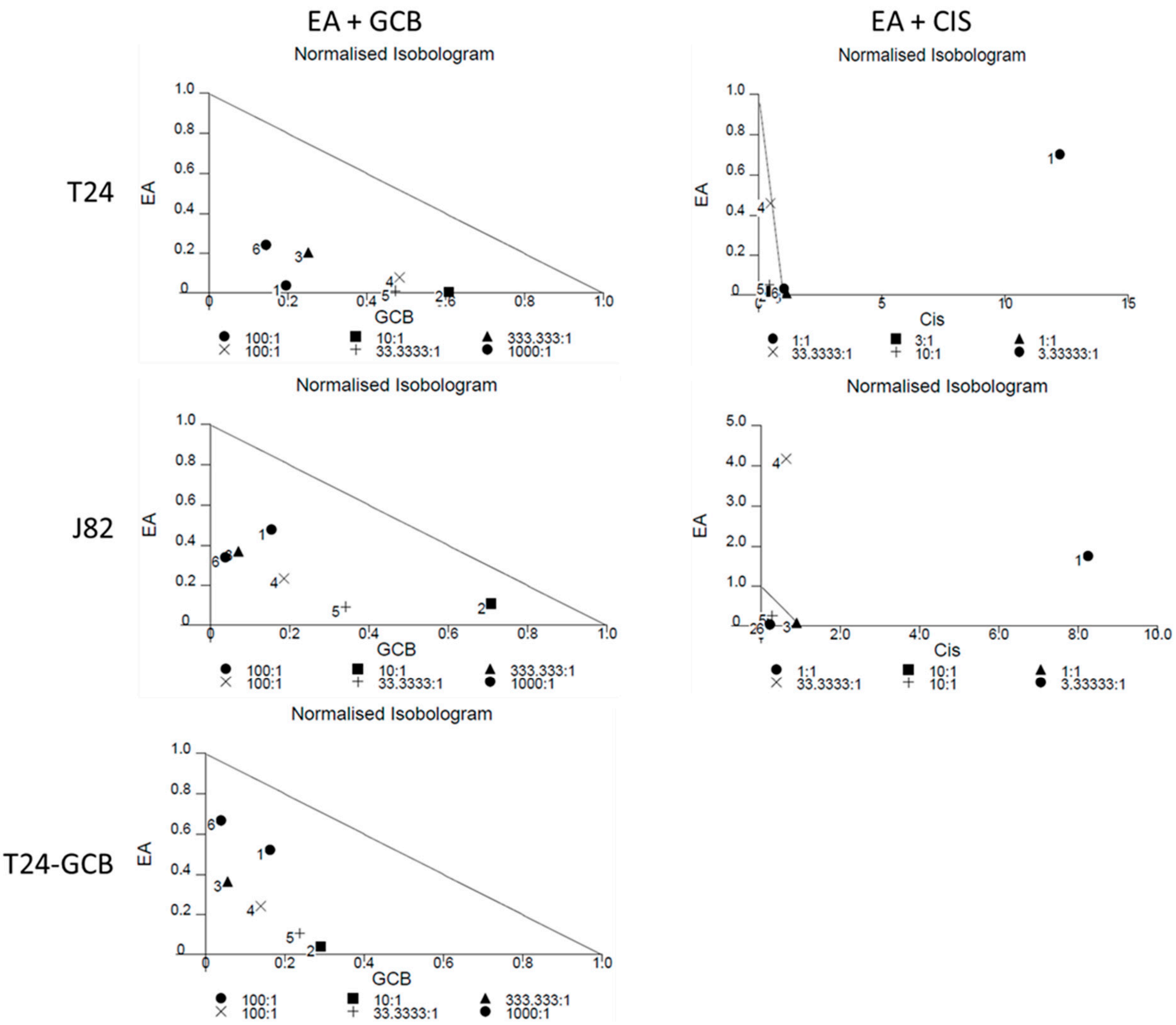

Figure S2. Isobologram analyses of combined effects between ellagic acid (EA) and gemcitabine (GCB) or EA and cisplatin (CIS) were compared in T24, J82 and T24-GCB cells.

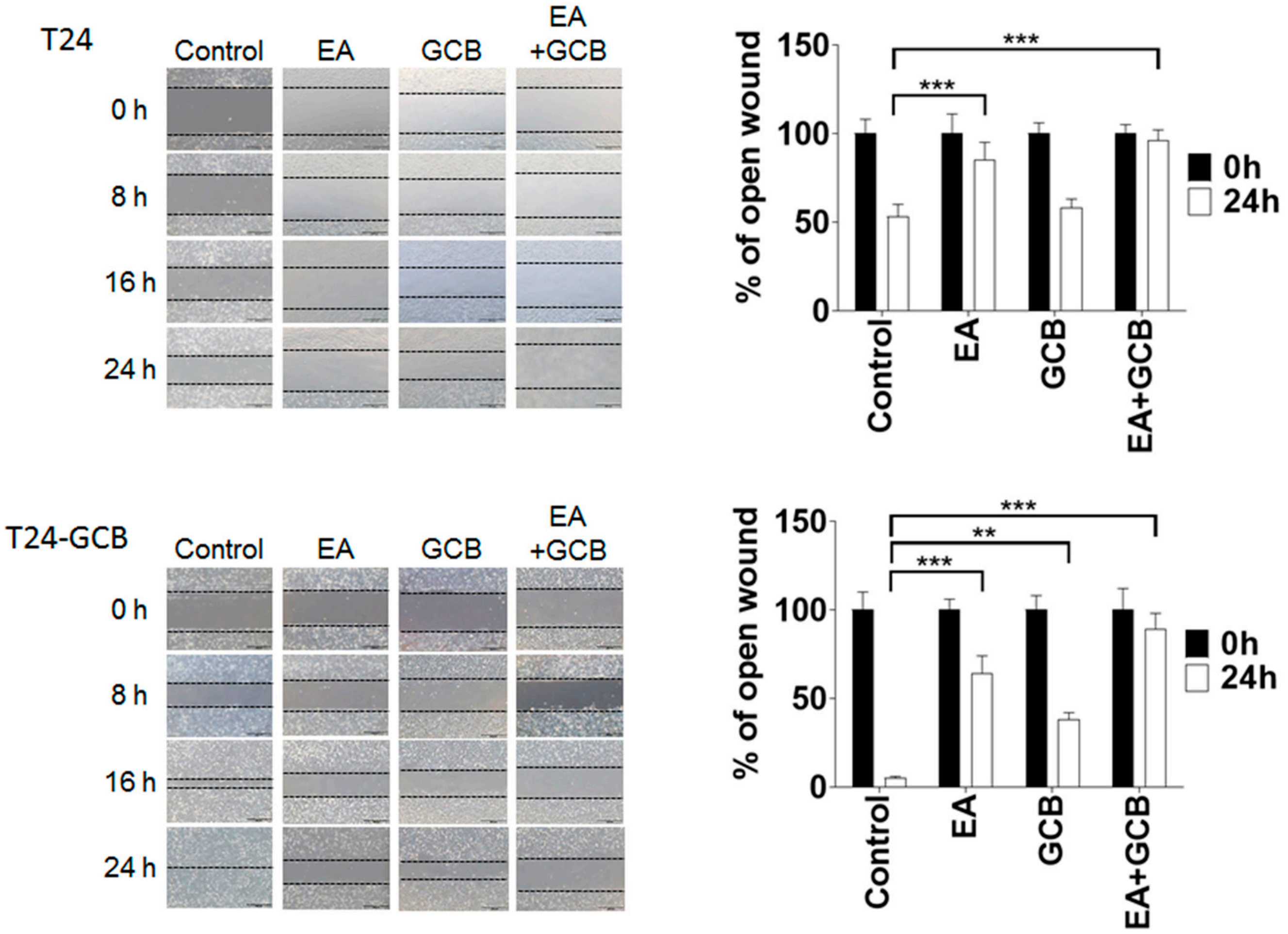

Figure S3. Cell motility of T24 and T24-GCB cells as determined by the wound-healing assay compared among the PBS control, EA, GCB and EA+GCB groups. Quantitative bar charts of migrated cells in the treated groups were showed on the right side. All statistical tests were analyzed with Student's t test with significance at ** <0.01 and *** <0.001.

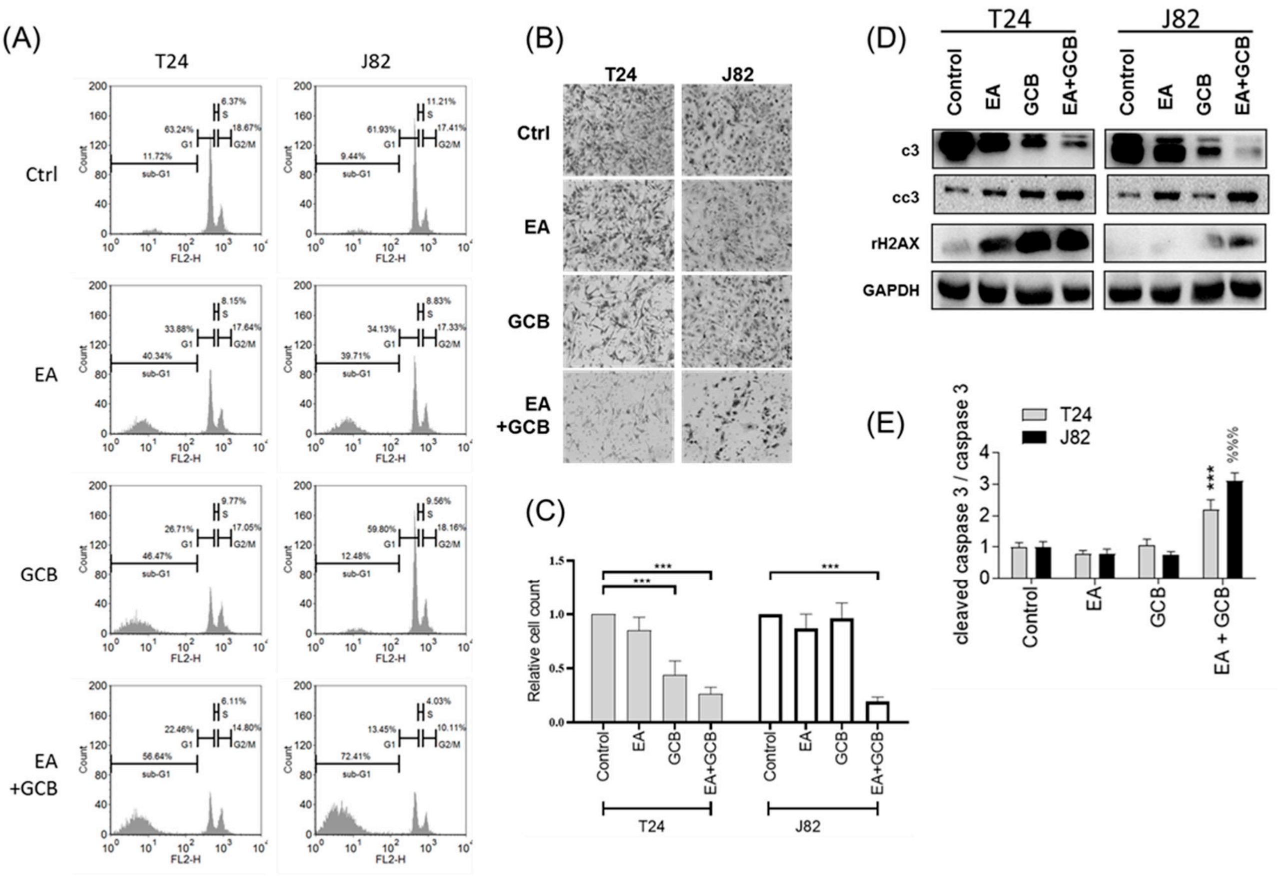

Figure S4. (**A**) Comparison of the cell cycle distribution of T24 and J82 cells among the PBS control, EA (30 μM), GCB (0.1 μM) and EA (30 μM) + GCB (0.1 μM) groups. (**B**) Results of the Matrigel-coated Transwell invasion assays compared among the same groups. (**C**) Quantitative bar chart of migrated cells among the treated groups. (**D**) Protein expression levels of procaspase 3 (C3) and cleaved caspase 3 (CC3) and γH2AX in T24 and J82 cells were compared among the same groups. (**E**) Quantitative bar chart of C3 and CC3 expression among the treated groups. All statistical tests were analyzed with Student's t test with significance at ** <0.01 and *** <0.001 (or %%% <0.001).

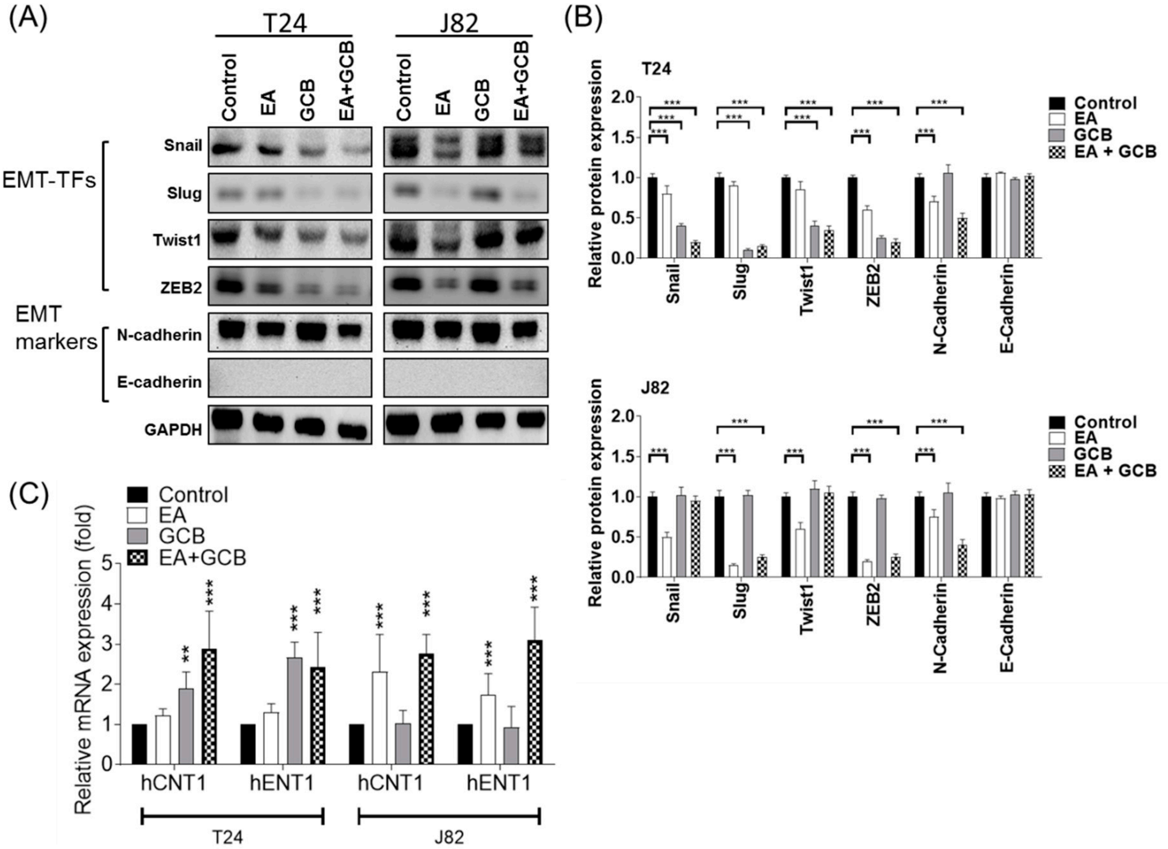

Figure S5. (**A**) Protein expression levels of EMT-TFs (Snail, Slug, Twist1 and ZEB2) and EMT markers (N-cadherin and E-cadherin) in T24 and J82 cells were compared among the PBS control, EA (30 μM), GCB (0.1 μM) and EA (30 μM) + GCB (0.1 μM) groups. (**B**) Quantitative bar chart of the protein expression of the indicated EMT-TFs among the treated groups. (**C**) Relative mRNA expression of hCNT1 and hENT1 compared among the same groups between T24 and J82 cells. All statistical tests were analyzed with Student's t test with significance at * <0.05, ** <0.01 and *** <0.001.

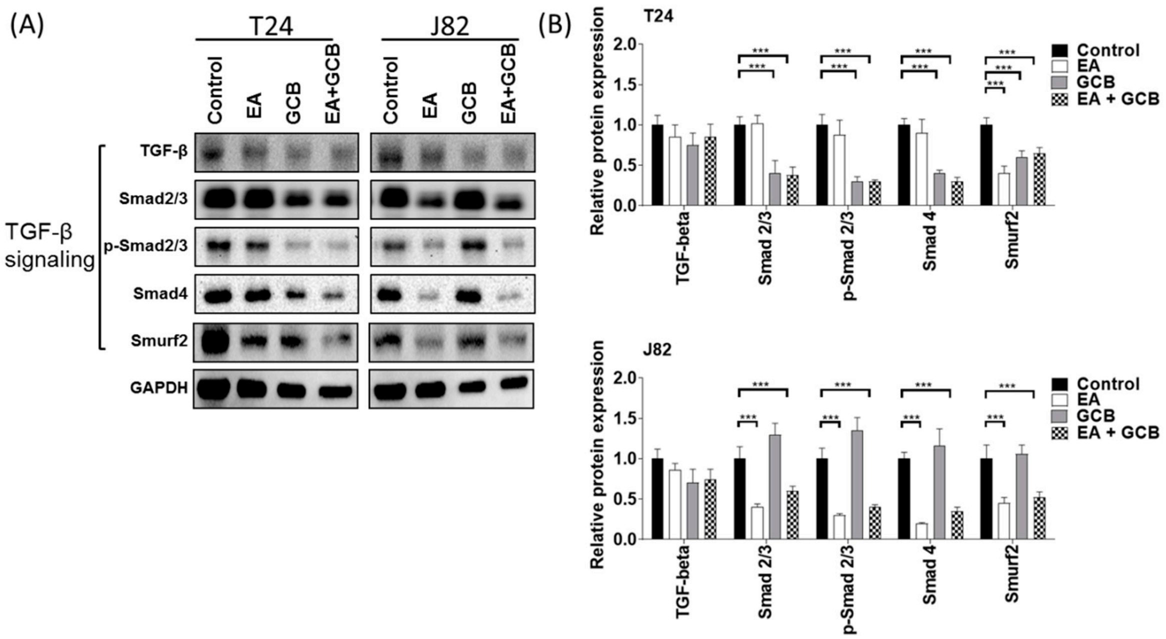

Figure S6. (**A**) Protein expression levels of members of the TGF-β signaling pathway (TGF-β, Smad2/3, p-Smad2/3, Smad4 and Smurf2) were compared among the PBS control, EA (30 μM), GCB (0.1 μM) and EA (30 μM) + GCB (0.1 μM) groups between T24 and J82 cells. (**B**) Quantitative bar chart of the protein expression of the indicated members among the treated groups. All statistical tests were analyzed with Student's t test with significance at * <0.05, ** <0.01 and *** <0.001.

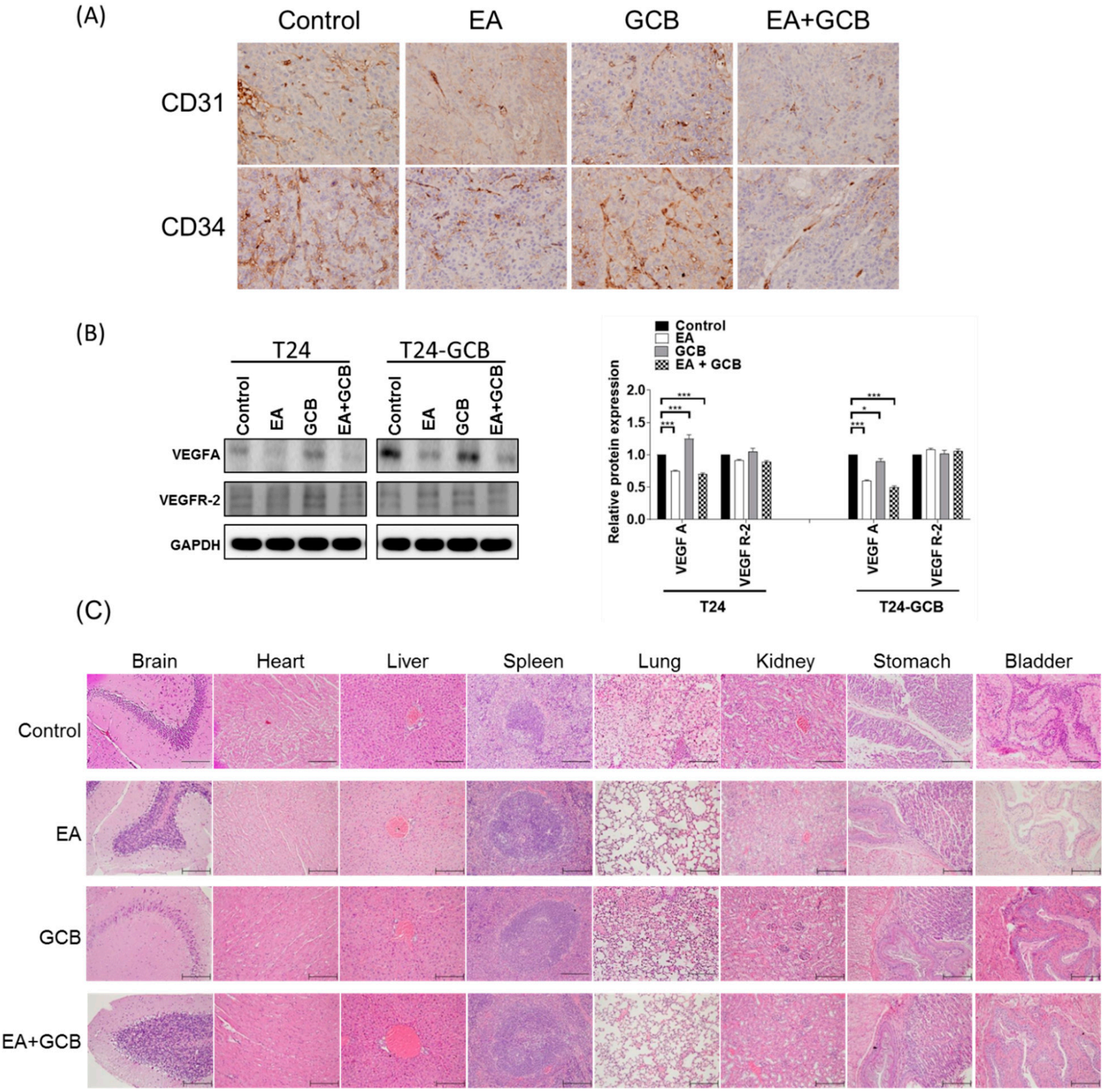

Figure S7. (**A**) Immunohistochemical staining of CD31 and CD34 in xenograft tumors from T24-GCB xenograft mice treated with the PBS control, EA, GCB and EA + GCB. (**B**) Protein expression levels of VEGFA and VEGFR-2 in T24 and T24-GCB cells compared among the PBS control, EA, GCB and EA+GCB groups. Quantitative bar charts of the expression of the indicated proteins among the treated groups were showed. All statistical tests were analyzed with Student's t test with significance at *<0.05, **<0.01 and ***<0.001. (**C**) Alignment of H&E staining of the brain, heart, liver, spleen, lung, kidney, stomach and bladder of xenografted mice that received the four treatments, and no perceptible necrosis was observed.

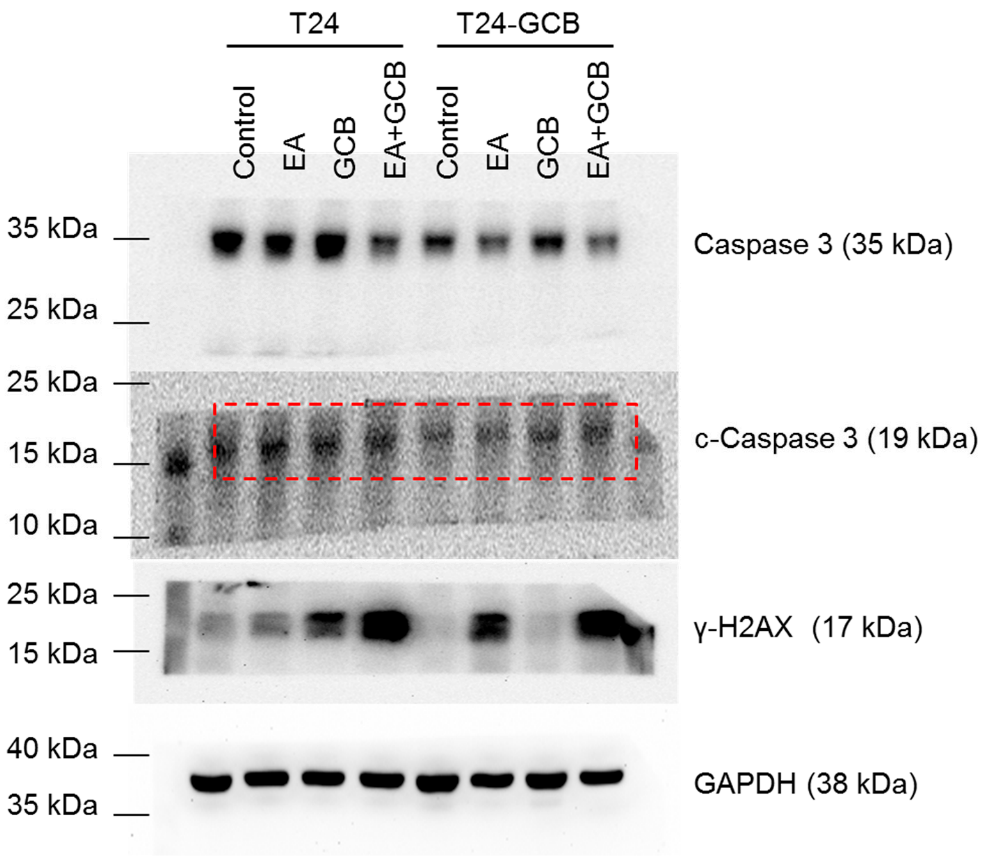
T24
T24-GCB
Control
EA
GCB
EA+GCB
Control
EA
GCB
EA+GCB
35 kDa
25 kDa
Caspase 3 (35 kDa)
25 kDa
15 kDa
10 kDa
c-Caspase 3 (19 kDa)
25 kDa
15 kDa
γ-H2AX (17 kDa)
40 kDa
35 kDa
GAPDH (38 kDa)

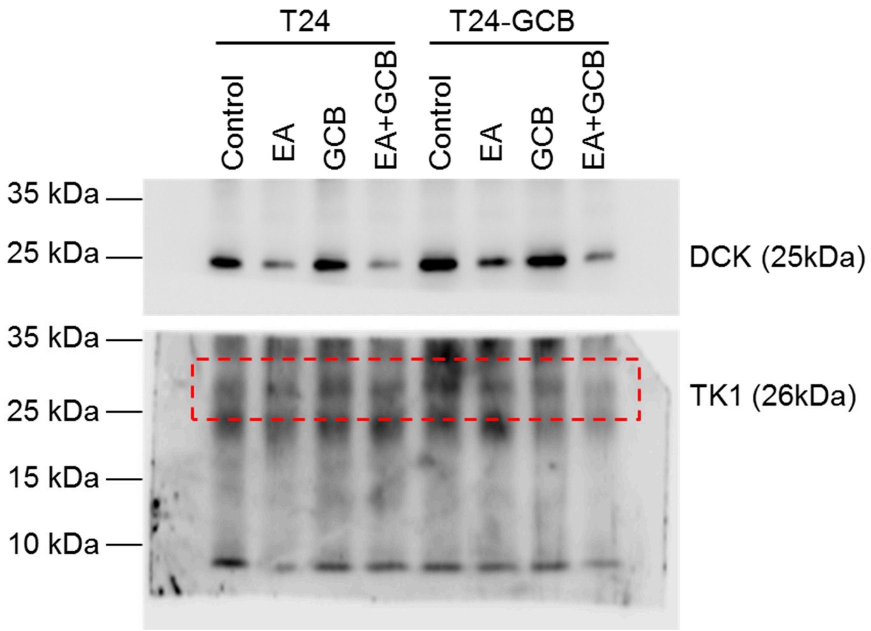
T24
T24-GCB
Control
EA
GCB
EA+GCB
Control
EA
GCB
EA+GCB
35 kDa
25 kDa
DCK (25kDa)
35 kDa
25 kDa
15 kDa
10 kDa
TK1 (26kDa)

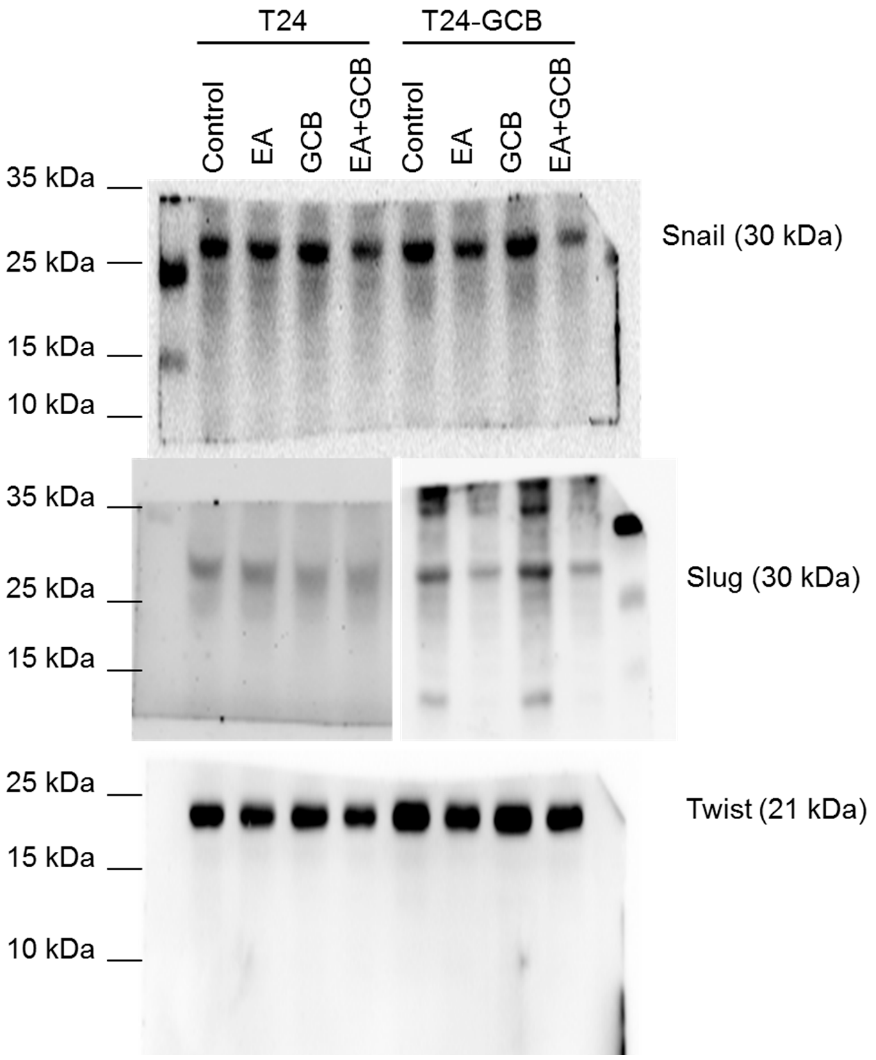
T24
T24-GCB
Control
EA
GCB
EA+GCB
Control
EA
GCB
EA+GCB
35 kDa
25 kDa
15 kDa
10 kDa
Snail (30 kDa)
35 kDa
25 kDa
15 kDa
Slug (30 kDa)
25 kDa
15 kDa
10 kDa
Twist (21 kDa)

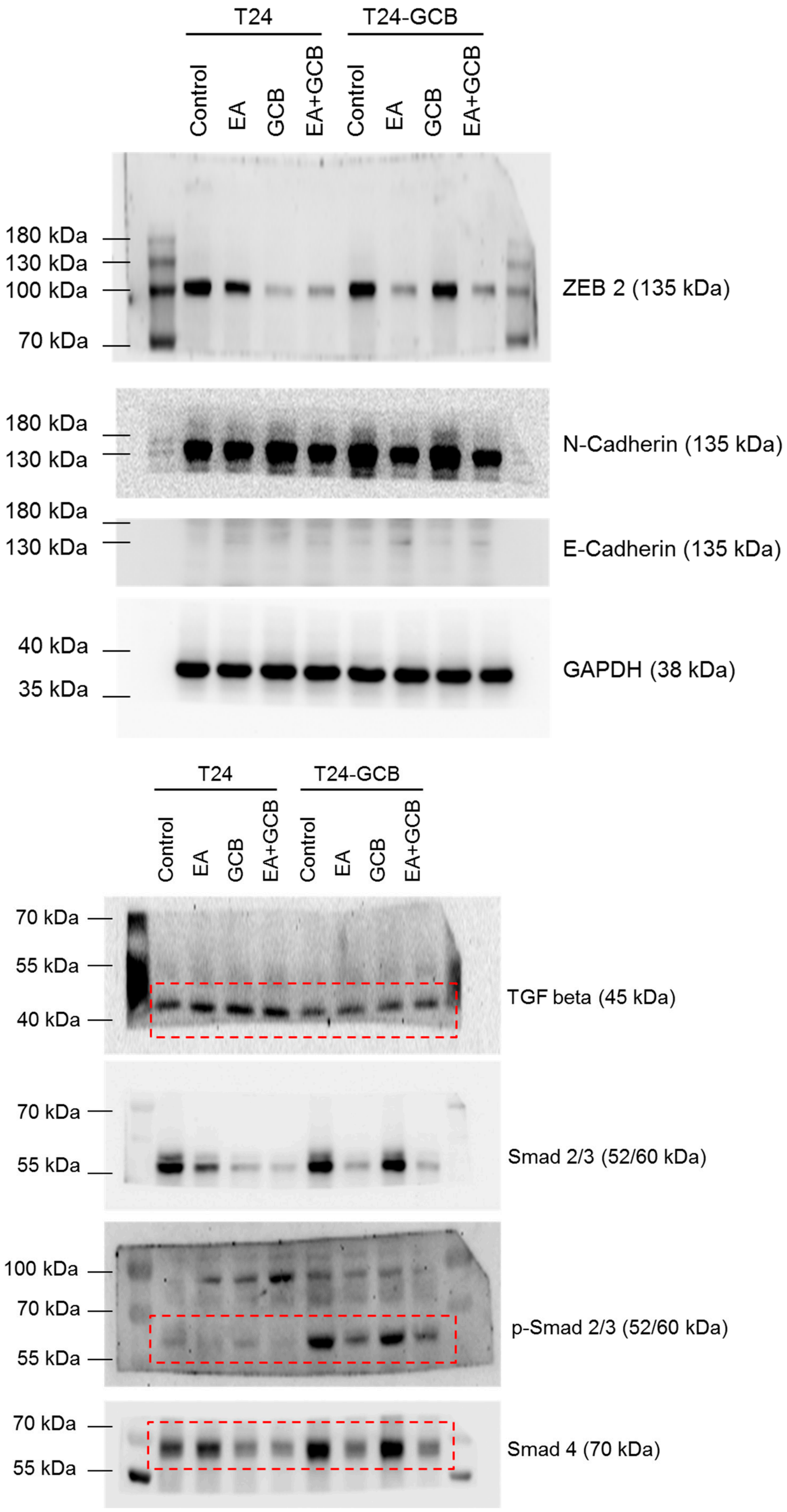
T24
T24-GCB
Control
EA
GCB
EA+GCB
Control
EA
GCB
EA+GCB
180 kDa
130 kDa
100 kDa
70 kDa
ZEB 2 (135 kDa)
180 kDa
130 kDa
N-Cadherin (135 kDa)
180 kDa
130 kDa
E-Cadherin (135 kDa)
40 kDa
35 kDa
GAPDH (38 kDa)
T24
T24-GCB
Control
EA
GCB
EA+GCB
Control
EA
GCB
EA+GCB
70 kDa
55 kDa
40 kDa
TGF beta (45 kDa)
70 kDa
55 kDa
Smad 2/3 (52/60 kDa)
100 kDa
70 kDa
55 kDa
p-Smad 2/3 (52/60 kDa)
70 kDa
55 kDa
Smad 4 (70 kDa)

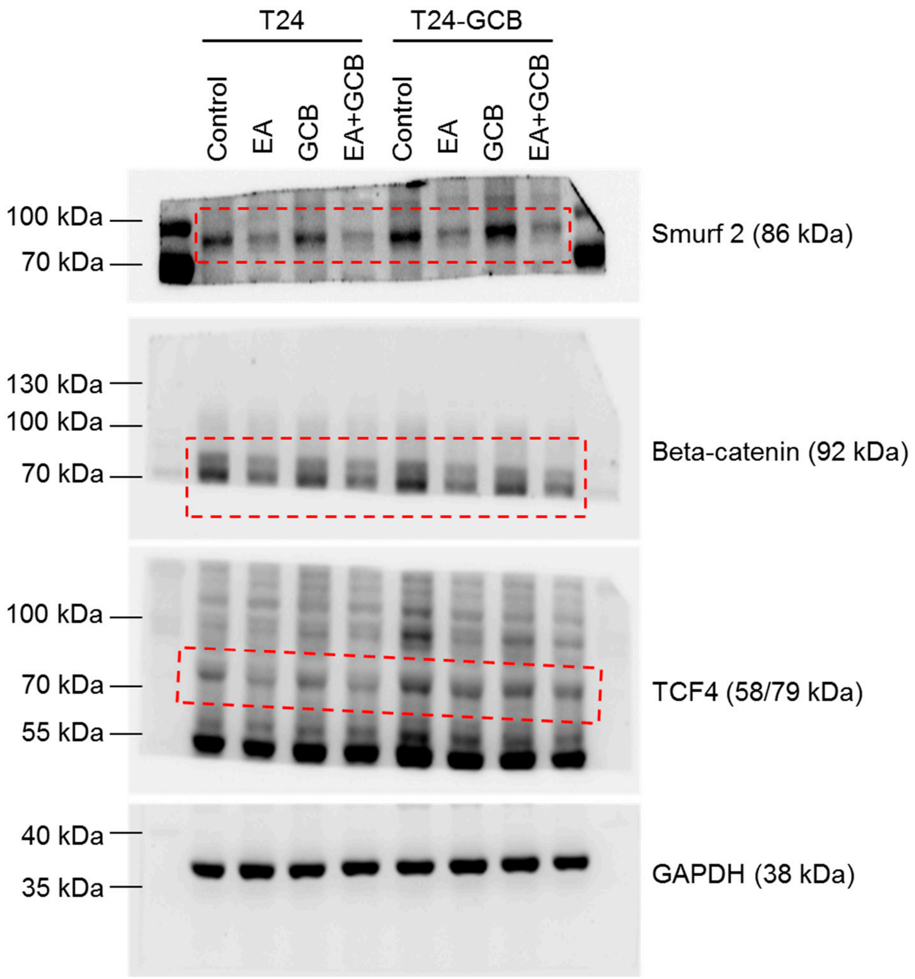
T24
T24-GCB
Control
EA
GCB
EA+GCB
Control
EA
GCB
EA+GCB
100 kDa
70 kDa
Smurf 2 (86 kDa)
130 kDa
100 kDa
70 kDa
Beta-catenin (92 kDa)
100 kDa
70 kDa
55 kDa
TCF4 (58/79 kDa)
40 kDa
35 kDa
GAPDH (38 kDa)

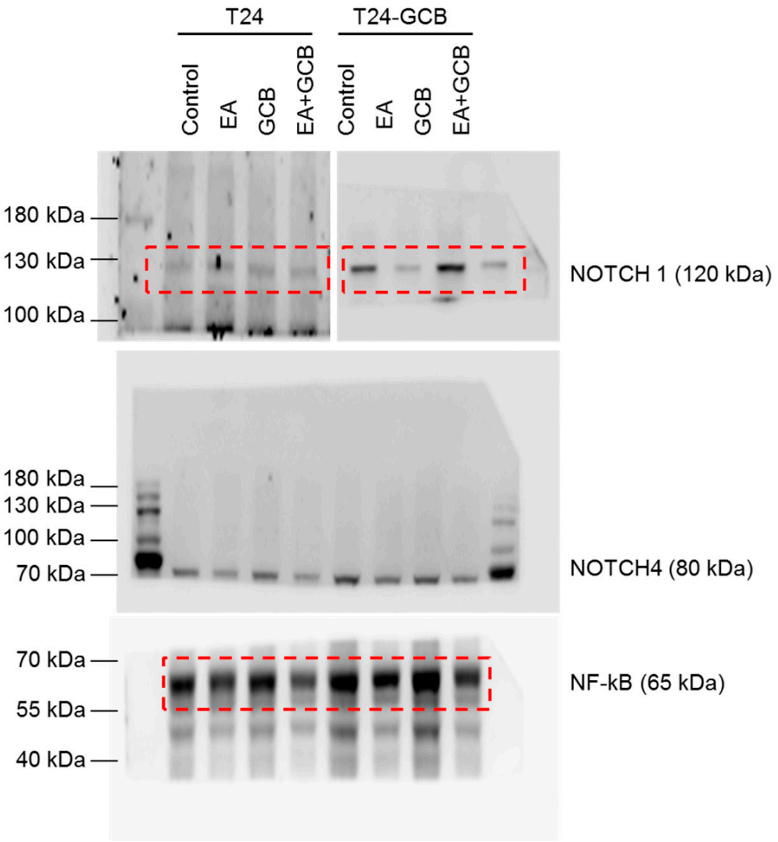
T24
T24-GCB
Control
EA
GCB
EA+GCB
Control
EA
GCB
EA+GCB
180 kDa
130 kDa
100 kDa
NOTCH 1 (120 kDa)
180 kDa
130 kDa
100 kDa
70 kDa
NOTCH4 (80 kDa)
70 kDa
55 kDa
40 kDa
NF-kB (65 kDa)

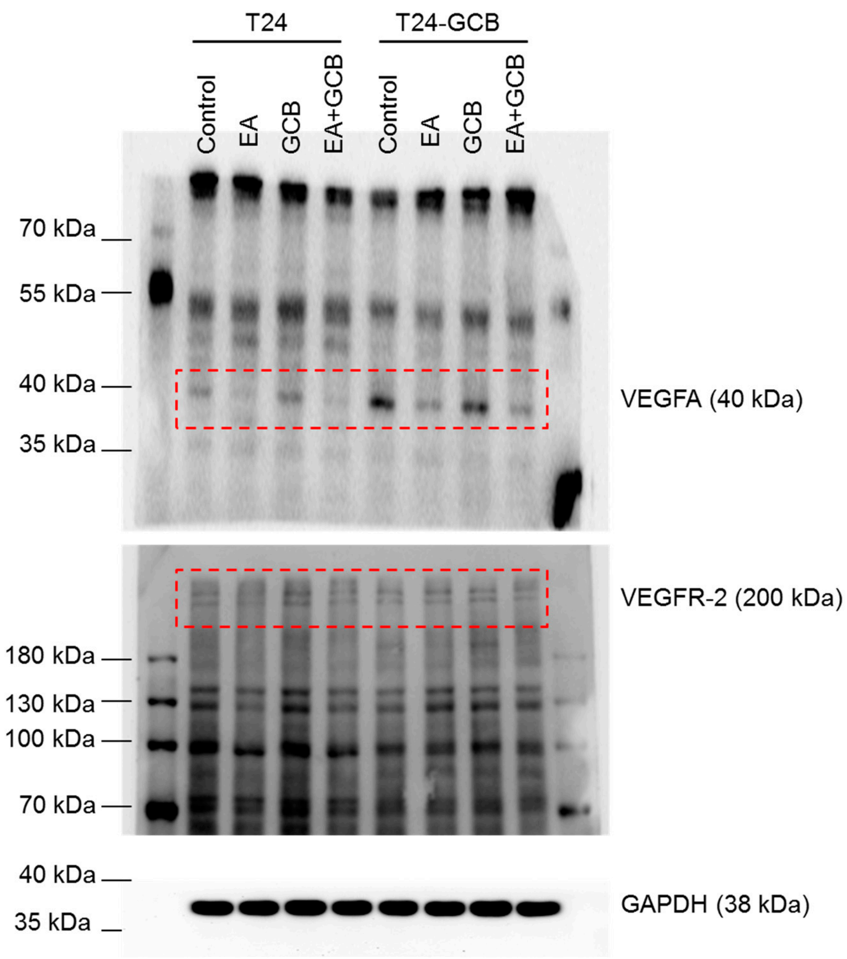

Figure S8. Original Western Blots.

Table S1. Lists of the primary antibodies.

Primary antibodies	Supplier	Cat. Number
caspase 3	Cell Signaling Technology	cat. no. # 9662
cleaved-caspase 3	Cell Signaling Technology	cat. no. # 9654
γ-H2AX	Cell Signaling Technology	cat. no. # 9718
dCK	GeneTex	cat. no. GTX632065
TK1	GeneTex	cat. no. GTX113281
snail	Abcam, Cambridge	cat. no. ab53519
slug	Abcam, Cambridge	cat. no. ab27568
twist 1	Abcam, Cambridge	cat. no. ab50887
ZEB 2	Cell Signaling Technology	cat. no. #97885
N-cadherin	Abcam, Cambridge	cat. no. ab18203
E- cadherin	Abcam, Cambridge	cat. no. ab231303
TGF-β	Abcam, Cambridge	cat. no. ab92486
smad 2/3	Cell Signaling Technology	cat. no. # 3102
p- smad 2/3	Cell Signaling Technology	cat. no. # 8828
smad 4	Abcam, Cambridge	cat. no. ab236321
smurf 2	Cell Signaling Technology	cat. no. # 12024
β-catenin	Cell Signaling Technology	cat. no. # 8480
TCF-4	Cell Signaling Technology	cat. no. # 2569
Notch 1	Cell Signaling Technology	cat. no.#3608
Notch 4	Cell Signaling Technology	cat. no. # 2423
NF-κB	Abcam, Cambridge	cat. no.ab231481
VEGF-A	Abcam, Cambridge	cat. no.ab1316
VEGFR-2	Abcam, Cambridge	cat. no.ab39256

GAPDH	Abcam, Cambridge	cat. no.ab9485

Table S2. Lists of the primer sequences.

Gene	Forward Primer	Reverse Primer
NT5C	GGACACGCAGGTCTTCATCTG	GCGGTACTTCTCACCCACACA
NT5C2	ACCTGCTGTATTACCCTTTCAGCTA	GCTCCACCGTTGATTCATGA
NT5C3A	AATCGGCGATGTACTAGAG	CATCTGCCATTCTTAAGTCTC
NT5M	CATCAGCATTTGGGAGTCAA	CAGCACAATCTGCTCCAGAA
dCK	AAACCTGAACGATGGTCTTTTACC	CTTTGAGCTTGCCATTCAGAGA
TK1	GGGGCAGATCCAGGTGATTC	GCATACTTGATCACCAGGCACTT
TK2	TTACCTTCGGACCAATCCTG	TGCTTCCGATTCTCTGGAGT
CDA	ATGGCCCAGAAGCGTCCTGCCTGCA	TCACTGGGTCTTCTGCAGGTCCTCA
DCTD	GTTGCCTTGTTCCCTTGTAA	TCTTGCTGCACTTCGGTATG
CMPK1	GGGCATATTCTTTGCTTCCA	TGCATTTCAAGGTTCCACTG
NME2	GTGCAGCGCGGCCTGGTGGG	GACCCAGTCATGAGCACAAGAC
DCTPP1	AAATGGACATCAACCGGCGA	AGTCACAGGGAATGTCCGCA
CTPS1	GTGGGCAAATACACGAAGTT	TCCTCGAACACCAAATCCTC
RRM1	TACCAACCGCCCACAACTTT	TCTCAGCATCGGTACAAGGC
RRM2	GACACAAGGCATCGTTTCAA	TCTATGGCTTCCAAATTGCC
hCNT1	TCTGTGGATTTGCCAATTTCAG	CGGAGCACTATCTGGGAGAAGT
hENT1	GCTGGGTCTGACCGTTGTAT	CTGTACAGGGTGCATGATGG
hENT2	ATGAGAACGGGATTCCCAGTAG	GCTCTGATTCCGGCTCCTT
